# Everyday activity strategies perceived by people with advanced cancer: a qualitative explorative study

**DOI:** 10.1186/s12904-025-01660-2

**Published:** 2025-01-30

**Authors:** Karen la Cour, Lisa Gregersen Oestergaard, Marc Sampedro Pilegaard

**Affiliations:** 1 Occupational Science, Department of Public Health, User Perspectives and Community-based Research, University of Southern Denmark, Campusvej 55, Odense M. DK, Odense, 5230 Denmark; 2 DEFACTUM, Central Region Denmark, Aarhus, Denmark; 3 Department of Public Health, Aarhus University, Aarhus, Denmark; 4 Department of Social Medicine and Rehabilitation, Gødstrup Hospital, Gødstrup, Denmark; 5 Clinical Department of Medicine, Aarhus University, Aarhus, Denmark

**Keywords:** Adaptation, Daily life, Routines, Functioning, Palliative rehabilitation, Advanced cancer, Patient perspectives

## Abstract

**Background:**

Despite growing research on the daily life of people with advanced cancer, more specific knowledge is needed about the specific strategies these people use to manage everyday activities.

**Purpose:**

This study explores how people with advanced cancer manage their everyday activities and describe their specific strategies.

**Methods:**

The qualitative study was designed with an explorative approach. Data from 28 people with advanced cancer was drawn from a trial including qualitative interviews to elicit participants’ perceptions about managing their everyday activities. Interviews were conducted in participants’ homes and analysed using an inductive thematic analysis.

**Results:**

Within an overarching theme of keeping ‘Daily life as usual’, the findings unfold participants’ specific strategies identified within two sub-themes ‘, Upholding routines’ and ‘Activity adaptations’. Upholding routines related to 1) Personal care and household and 2) Leisure-, social- and work-life. Activity adaptation about 1) Working with and around physical limitations; 2) Sharing, delegating, and letting go; and 3) Enlisting ‘outside’ support.

**Conclusion:**

This study specified participants’ specific and distinct self-developed strategies within routines and activity adaptations. The strategies reflect participants’ needs for maintaining functioning while relieving pain and mourning, which holds essential potential for informing person-centred intervention development integrating rehabilitation in palliative care.

## Background

People with advanced cancer strive to be involved and actively contribute to daily life [1–5]. Doing so can convey a sense of normality, autonomy, and dignity despite ongoing losses of function and declining health [6–8]. Therefore, several studies have examined the daily lives of people with advanced cancer, focusing on their everyday activities [7, 9, 10]. Performing everyday activities is a crucial indicator of how people experience their illness and achieve quality of life [6, 11–13].

A qualitative study examined the strategies people with advanced cancer use when performing everyday activities. The study found that lowering one’s expectations enabled participants to continue performing familiar activities, which offered experiences of competence [1]. An explorative mapping study found that people with advanced cancer, while dealing with their symptoms and cancer treatment, strive to establish continuity and compose rhythms of routine and novel activities in daily life [7]. This is supported by a recent longitudinal study of working-aged adults living with advanced cancer [14]. They found that despite disrupting routines and daily life, people seek to continue doing what is important to them in modified ways involving ongoing adaptational processes through engagement in activities [14].

Although several studies have explored everyday activities, including the adaptational processes of people with advanced cancer [3, 6, 7, 14–16], hardly any studies are specific about what distinct strategies these people use. Hence, this study aimed to explore how people with advanced cancer manage their everyday activities and describe their strategies.

## Methods

### Design

The present study draws on data from a larger randomised controlled trial (RCT) involving 242 adults with advanced cancer living at home [17]. The RCT examined the effectiveness of the Cancer Home-Life Intervention in terms of performance, participation in everyday activities, and health-related quality of life [18, 19]. As part of the study, the present sub-study was designed with an explorative qualitative approach providing insights into participants’ everyday strategies. Interview data were collected at baseline and collected throughout 2016 [17, 20]. The study is reported following the COREQ checklist [21].

### Participants

A total of 242 adults living at home were recruited from outpatient clinics at two Danish hospitals and included participants with a cancer diagnosis considered incurable by the responsible oncologist and with a score of 1–2 on the WHO performance scale, indicating difficulties performing physically strenuous activities [22]. For the present study, a random sample of 28 participants (17 women and 11 men) with a median age of 69 was drawn. The random process involved selecting every eighth participant from the total list of included participants. Table 1. shows participant characteristic details.

**Table 1:** Participant characteristics (*N* = 28)

				Study population (*N* = 28)	
**Demographic**					
Age (years), median (IQR)			69 (61;74)		
Women, n			17		
Living with a partner, n			20		
Receiving chemotherapy, n ^*^			23		
Occupational status, *n*					
Working			5		
Retired			21		
Sick leave			2		
Education, n					
≤ 10 years			4		
11–12 years			8		
> 13 years			16		
Primary tumour site, n (%)					
Gastrointestinal			8		
Lung			6		
Breast			7		
Prostate			5		
Bladder			1		
Gynaecological			1		

IQR: interquartile range

^*^Two participants had missing values

### Data collection

All participants were subject to a brief qualitative interview conducted in their homes as the upstart of a more in-depth examination of managing their everyday activities, which was used for the larger RCT study [18]. The qualitative interviews were conducted before randomisation, piloted before the study began, and proved useful for gaining insights into subjective perceptions of daily life. The interview was initiated with the open-ended question: “I would like to hear how you manage your daily life and its activities”. This question elicited participants’ perceptions of their everyday activities, concerns, challenges, and strategies. The interviews were conducted by occupational therapists trained as data collectors and ranged from 10 to 20 min, which were audio-recorded and transcribed verbatim.

### Data analysis

The interview data were analysed using an inductive thematic methodology, which involved reading and re-reading transcripts and identifying themes and sub-themes [23, 2, 24]. Particular attention was paid to the participants’ perceptions of how they managed their everyday activities and what they did to do so. Emerging themes were refined through discussions between the first and the last author and a senior researcher who contributed to the early work of this study. Continuous reflective debriefing of the transparency between data and identified themes among the authors ensured the rigour of the analysis.

We identified an overarching theme of living ‘Daily life as usual’, elaborated through two interrelated sub-themes of activity strategies related to upholding routines and activity adaptation. To elaborate more detailed analysis within these sub-themes, we identified routines related to specific activities and distinct forms of adaptation.

## Results

The findings demonstrate the overarching theme of living ‘Daily life as usual’ through which specific and distinct strategies were identified within two interrelated sub-themes: A) ‘Upholding routines’ and B)‘Activity adaptation’. (A) ‘Upholding routines’ was related to 1) personal and household care and 2) leisure, social and work life. (B) ‘Activity adaptation’ was related to 1) Working with and around physical limitations, 2) Sharing, delegating, and letting go, and 3) Enlisting ‘outside’ support (See Table 2. for an overview).

**Table 2:** Strategies for upholding routines and activity adaptations

Activity adaptations			Upholding routines			
Working with and around limitations	Shifting responsibilities	Enlisting external support	Personal care and household	Leisure	Social life	Work life
New routine Reducing Prioritizing Pacing Breaking up	Stopping Avoiding Delegating Sharing	Employer, colleagues Municipality or private Social networks Neighbor Extended	Personal hygiene Sleep Rest Cooking + baking Cleaning (light) Cleaning (heavy; hoovering) Laundry Shopping (daily, light) Shopping (weekly, heavy) Garden maintenance	Leisure, knitting, sewing, family research walking biking	Socialising Family Friends Social network	Work

The themes are presented separately below for the sake of clarity. Quotes are anonymised and used paradigmatically as they demonstrate exemplary perceptions of specific and distinct strategies shared among the participants.

### Daily life ‘as usual’

A predominant strategy among participants related to the overarching theme of ‘Daily life as usual’ includes a broad range of everyday activities related to the contexts of participants’ homes and family lives and work for those still maintaining work while living with advanced cancer (*N* = 5). These foci reflect the question posed, the broader context of the study, the participants’ advanced stage of cancer, and the fact that most participants are retired (*N* = 21) or on sick leave (*N* = 2).

The participants frequently stated that they carried on their everyday lives ‘as usual’. This appeared to be driven by a determination to ‘do as much as possible oneself’ (woman, 75) and reinforced by the belief that it is ‘important to stay active’ (woman, 77). Such statements highlight the centrality of activities in maintaining daily functioning and underscore the strategies for accomplishing them despite increasing limitations and ongoing losses.

To maintain everyday life ’as usual,’ the participants described upholding routines in everyday activities, i.e., one of the women shared:

The more we live as usual, the better; that’s how we feel.

### Upholding routines

The routines were upheld in regard to specific activities; (a) personal care and household and (b) leisure, social, and work life (Table 2).

#### Personal care and household

The participants mainly focused on recounting some of the many daily domestic chores they carried out.

As a woman explained:

I get up around 8 am, have breakfast, shower and if vacuuming.

and dusting is needed, then I do that and wash clothes and enjoy the weather.

for the moment and other days when I might not do so much,

then I read and watch TV or am on the PC.

By preparing breakfast in the morning, cleaning, and tidying living spaces daily, this woman used activities to uphold daily routines.

Regarding the cleaning activities, we found that the participants mainly did them except for hoovering. When challenged, they seemed to seek other strategies to influence and maintain some sense of control over how such routine activities were carried out. Those who did not manage domestic activities ‘as usual’ due to ill health appeared to attend to domestic chores within their range of capabilities and physical resources.

Another aspect of upholding routines was expressed regarding personal needs and hygiene, such as dressing, washing, and toileting. The emphasis was on maintaining a clean appearance despite serious illness.

A male participant shared:

I get up depending on how long I have been awake during the night. I get up around 7–8 a.m. when the sun shines through my bedroom window. Then I make coffee and have a small whisky—yes, yes—and then, of course,…… if there is a spot on my shirt after shifting the stomia, I put it in the washing machine, so I am clean—clean shirt and stomach.

Managing these intimate aspects appeared to be a ‘measure’ of routine activities used by many participants to indicate that their daily lives carried on ‘as usual’ by still being capable.

The findings revealed that the participants strove to maintain daily domestic and personal hygiene activities as routines, providing the sense of ‘as usual’, if possible.

#### Leisure-, social- and work-life

To illustrate how many of the participants were upholding routines through leisure-, social- and work-life, a female participant said:

I sew and knit in front of the TV, attend choir, and organise a social club at the parish house every other Thursday. I also volunteer at the church and help with baking for such events. What else do I do? Yes, we attend sports on Thursday mornings and play volleyball.

The participants’ leisure activities were mainly sedentary: watching TV, reading the newspaper, knitting and sewing (women), computing, researching family history (men), and stamp collecting (men). Exercise-orientated leisure activities included arranged routines of walking with a partner or neighbour, walking the dog, going upstairs or downstairs, and using a stationary exercise bike. Furthermore, for most of the participants, treatment, such as receiving chemotherapy or attending regular physiotherapy sessions, had become recent additions to their routines.

As one of the male participants said:

It’s pretty regular. I attend training twice a week with a physiotherapist, and for the past two months, I have not gone for chemo; otherwise, that occupies every two weeks, so that is how it was earlier.

Daily social activities and contacts focused primarily on live-in partners and adult children (and grandchildren) who lived locally. In addition, regular contact with adult children included help with weekly shopping, family visits and outings, and most participants mentioned the occasional gathering with friends (often in participants’ homes). Others shared that medical appointments and treatment limited their ability to be active and socialise, especially after chemotherapy.

The participants who were working and those who were engaged in volunteer work stressed the importance of their work, volunteer tasks, and professional contacts. They valued the social relations, support, and understanding they received from colleagues and managers.

A male participant said:

I want to work. I have an appointment with my boss, who understands my situation, so I try to go to work as often as I have the energy.

Upholding some kind of working routine underpinned the everyday lives of mainly working participants. All had reduced working hours, often a few hours every day, to accommodate limited stamina; such altered working patterns called for new daily routines while also being amenable to ongoing decline.

The findings of strategies for upholding daily routines revealed that participants’ prioritisation of activities tended to be grounded in slightly gendered patterns of household and domestic activities for women and personal care and gardening for men. Working participants, with either professional or volunteer tasks, seemed to attend to their professional rather than domestic responsibilities.

Through these ways of carrying on with specific activities, the participants’ daily life can continue ‘as usual’, with the upholding routines used as an essential strategy.

### Activity adaptations

To live their lives with advanced cancer ‘as usual’ while adapting to gradual losses, the participants developed diverse and distinct strategies, which were elaborated through (a) working with and around physical limitations, (b) sharing, delegating and letting go, and (c) enlisting ‘outside’ support.

#### Working with and around physical limitations

Many participants mentioned struggling with fatigue, and most seemed acutely aware of their reduced energy and strength due to advanced cancer and treatments. This resulted in common adaptive strategies, including pacing oneself (slowing down), breaking activities into smaller units, resting between and forging new activities. Most participants mentioned pacing themselves.

As a female participant shared:

I manage daily life reasonably well, I think, even though it takes time – right from cleaning the windows, which I did the other day, and I was also down on all four scrubbing the floor, so that is fine, so I take a few brakes when I vacuum; that is the most demanding, to vacuum.

Participants described breaking domestic activities into smaller units and resting between each step, for example, hoovering room by room rather than considering hoovering the whole apartment or house as one task. Likewise, leisure activities involved pacing oneself, such as walking at one’s own pace, knitting for shorter periods, adapting to bodily abilities by reducing a familiar walking route, or simply sitting and resting on the veranda when walking any distance became impossible.

Some participants developed new ways of managing needed activities to accommodate physical limitations. As a male participant shared, he and his wife found it difficult to carry heavy shopping for any distance. To minimise distance, he described driving the car as close to the front door as possible to unload the shopping before parking further away. Others carried out meal preparation or personal hygiene activities while sitting down rather than standing. In addition, certain valued activities may cause symptoms or effects requiring remedial action. One woman described that tidying her home in the morning caused her bodily pain. She, therefore, took painkillers after having finished her domestic activities and rested for a while to overcome the remaining day. However, needing more rest and sleep may not always feel comfortable or easy to do. Another woman, for example, disclosed that she rested as soon as her partner left the house, thus disguising the toll living with advanced cancer has on her.

Others maintained activities depending on their energy, strength, and well-being. A male participant described a ‘good day’ as ‘perfect with energy; by contrast, a ‘bad day’ was characterised by being in bed all day. Thus, burdening symptoms, side effects, and the demands of hospital appointments impacted the level and kind of activities participants could carry out and how they could do so.

With declining energy and well-being, most participants appeared to have reduced social interactions. Their strategy of pacing themselves came to the fore, and planning activities adapted to their declined energy. Participants shared that attending family gatherings could be tiring, hence using strategies such as leaving early before becoming too tired, having friends visiting at home to save having to make a journey and reducing working hours and spreading events over several days. Those in work further mentioned having little energy for socialising beyond the immediate family or for domestic activities, which they tended to outsource.

#### Sharing, delegating, and letting go

These strategies were characterised by subtle shifts in responsibilities away from the person living with cancer. Notably, certain lighter household activities became shared undertakings, such as preparing meals together and jointly going shopping, whereas physically demanding activities may be delegated to others. In this way, different activities became ‘re-allocated’ over time. Division or delegating between genders was also identified, which is noticeable with heavy domestic chores, such as laundry or hoovering. Such activities that required physical strength and energy were either shared with a partner or delegated to them or someone else.

As a participant said:

One does all kinds of things in a home: washing floors, cleaning the bathroom, and shifting the bed linen. We share these tasks, and we also share grocery shopping. Because we have no local store, we typically drive to it together.

Some women shared that they used to garden but no longer had the strength and energy to do so, leaving all gardening to their partners. However, garden maintenance also required physical strength, and several men mentioned their inability to manage heavy garden activities. Still, the male participants seemed to have had less opportunity to delegate such physically demanding activities to their female partners (if they were partnered), as their partners tended to be elderly.

A strategy mentioned by some of the women, rather than completely giving up responsibilities for domestic chores, ‘instructed’ or ‘supervised’ their husbands/partners. Other participants deliberately avoided physically demanding activities and eventually let go of them once they became too demanding, especially heavy shopping, gardening, or cleaning the car.

As a male participant told us:

There are certain things (activities) that I must let go of and stop doing, which I would like to do but don’t get to immediately. There are probably some things I avoid that I used to do and would like to do, but it just takes some more time, and it does not get done as I would like.

Or, as he also pointed out, lowering the expectations for how soon things are done and to what quality. Also sharing the losses he mourned.

#### Enlisting ‘outside’ support

Most participants in this study lived with a partner, who, over time, seemed to be taking on activities that became too challenging for the person with cancer. However, couples and people who lived alone enlisted or accepted support beyond their primary household unit. Most commonly, adult children, especially those living nearby, stepped in to do the weekly shopping, look after the garden, or drive a parent to appointments. Some participants received domestic help through the local municipality or privately employed a cleaner.

A female participant said:

I receive cleaning every two weeks, and then I have a small Hoover, which I can use for most of the two weeks.

A male participant shared:

I am not a chef in cooking, but the fishmonger comes on Wednesdays, and then I go down and buy a couple of smoked herrings.

These findings indicate an overall progression from initially working with and around physical limitations to sharing and delegating activities, eventually discontinuing some activities and enlisting ‘outside’ support, mainly if activities required strength and were physically demanding.

## Discussion

This study explored how people with advanced cancer manage their everyday activities and describe their specific strategies. The findings showed a striving to live daily life as usual among the participants, managed through a strategy interplay between, on the one hand, upholding routines through everyday activities and, on the other hand, self-developed distinct activity adaptations to ease the progressive limitations imposed by ill-health. In other words, the study identified detailed individual descriptions of specific and distinct strategies to maintain functioning, compensate, and alleviate the mourning of ongoing losses.

The study enhances our understanding of ways in which to support people with advanced cancer by identifying the specific strategies they use to adapt their activities in response to limitations. These strategies are distinct and separable from one another, as they describe details like shifting responsibilities and seeking external support, which can be categorised as compensatory, additional, and substitutional. They are characterised by their individualised use depending on the participant’s health and life context and may change over time to accommodate physical decline. Morgan et al. refer to contending with ongoing deterioration as ‘the work of adaptation’ and suggest that this occurs ‘through struggling to participate in everyday occupations’ [3]. Our findings complement that study by not only highlighting the everyday activitiess but also specifying the distinct ways participants negotiate and conduct everyday activities.

The study further shows that the activities participants predominantly sought to uphold in their everyday routines were related to structured daily routines, reflecting diverse understandings of normality. These results relate to earlier studies, which also noted that people with advanced cancer seek to live life as usual [4, 7, 15, 25]. In extension, the present findings demonstrate how enabled perceptions of daily life ‘as usual’ range from managing personal hygiene to maintaining a domestic life of household chores or ongoing social and professional involvement.

The increasing functional limitations imposed by advanced cancer, significantly pronounced symptoms of fatigue, impacted participants’ everyday activities, often reducing activities to home-based sedentary undertakings, as also found in prior studies [6, 15]. Sedentary pursuits, however, do not imply that participants were not active [3]. Instead, participants’ perceptions indicated that they abandoned physically demanding activities in favour of less taxing pursuits to prioritise available energy into activities that supported their identities and everyday autonomy. Strategies that entail a gradual shifting of activities and responsibilities to others also serve a continuation of life ‘as usual’ in the home, whereas procuring external support may herald a need to let go of particularly demanding activities, which may not only challenge a sense of self or personal autonomy but also contest everyday life ‘as usual’. Thus, strategies of working with and around functional decline and losses may compensate for particularly physical limitations while maintaining one’s sense of self and daily life ‘as usual’.

We identified potential gender differences in strategies; for example, male participants were sometimes unable to delegate heavy work to their spouses. However, our study does not provide sufficient data to tease out specific gendered strategy patterns. Exploring gendered strategies was not an aim of the present study but certainly suggests a topic to pursue in future studies.

The findings in this study bring attention to the personal and creative ways in which people with advanced cancer develop distinct strategies, reflecting the resources people possess. However, some of these self-developed strategies may sometimes contradict what health professionals find beneficial and effective [6]. This is illustrated in the study by Peoples et al., who found that strategies that enabled the participants to manage specific everyday activities were sometimes counterproductive, for example, by being strenuous and tiring [6]. Also, in our study we found that participants would take medication or rest as soon as they could to compensate for the tolls of doing daily chores. On that basis the insights into the participants’ self-developed strategies may be helpful for health professionals to both support functioning and provide alleviation in the conduct of everyday activities. Moreover, providing supportive interventions for people with advanced cancer can benefit from building upon and integrating functional aspects of rehabilitation into palliative care and thus acknowledge the potentials of palliative rehabilitation.

### Strength and limitations

A strength of this study is the detailed insights into specific strategies related to structural and routine divisions of activities, including the distinct activity adaptations identified particularly in this population. Although we only used a single open-ended question to elicit the qualitative interviews, which may be a limitation, this approach opened for rich and detailed insights as the participants unfolded detailed information about the specific ways they managed daily activities. Moreover, the open-ended approach seemed to invite and allow participants to speak freely and expand their perceptions.

## Conclusion

Our study shows that managing everyday activities for people with advanced cancer is driven by intentions to maintain daily life as usual. This is done by developing deliberate strategies of upholding routines and using specific activity adaptions that are distinctly separable. The findings reflect people’s immense resources for self-developing strategy, which should be considered when developing interventions to support people in their best possible daily lives and build upon individual and shared capacities. Collectively, this can inform the nuanced development of person-centred interventions integrating rehabilitation in palliative care for people living with advanced cancer.

## Data Availability

Data are saved on a secure server at the University of Southern Denmark. The datasets generated and analysed during the current study are not publicly available due to Danish data protection law.
